# Psychometric Network Model Recovery: The Effect of Sample Size, Number of Items, and Number of Nodes

**DOI:** 10.3390/ejihpe15110235

**Published:** 2025-11-18

**Authors:** Marcelo Ávalos-Tejeda, Carlos Calderón

**Affiliations:** Escuela de Psicología, Facultad de Humanidades, Universidad Católica del Norte, Antofagasta 1240000, Chile; ccalderon@ucn.cl

**Keywords:** psychometric networks, gamma hyperparameter, sample size, number of variables, EBICglasso

## Abstract

In recent years, network psychometrics has emerged as an alternative to the reflective latent variable model. This model conceptualizes traits as complex systems of behaviors mutually interacting with each other. Although this model offers important advantages compared to the reflective model, questions remain regarding the necessary sample size and the influence of factors such as the number of nodes and edges. This study aims to evaluate the psychometric network model performance under different conditions of sample size, number of nodes, and number of edges. The methodology involved a simulation with 1000 replicates for each combination of sample size, number of nodes, and the value of gamma parameter, which is used to determine the magnitude of the edges considered significant. The effect of these conditions on the accuracy of edge estimations and centrality indices (strength and expected influence) was assessed using sensitivity, specificity, and bias indicators. Results suggest that sample size and network complexity have a more significant impact than *γ*, methodological guidelines being proposed to support decision-making in applied research. In summary, this study provides empirically grounded recommendations that can guide applied researchers in designing robust psychometric network analyses and ensuring reliable estimation of model parameters.

## 1. Introduction

In social and health sciences, the measurement model traditionally used is the reflective latent variable model, which assumes the existence of continuous latent variables explaining item variations by means of a regression function. Assuming this model involves making decisions concerning the design of measurement instruments and also the procedures to assess them. These decisions are based on assumptions such as latent variable distribution, local item independence, and measurement error independence, among others. Many of these assumptions cannot be empirically contrasted and/or imply the use of restrictions that make models little realistic or blur the possible complexity of the latent trait.

In the past few years, network psychometry has emerged as an alternative model, resulting from the application of the network theory to psychological instruments. Unlike the traditional model, covariations among items are not explained by a latent trait. On the contrary, the trait is considered as a complex system of observed indicators mutually influential and interacting (Cramer et al., 2010; Schmittmann et al., 2013; Van Der Maas et al., 2006).

### 1.1. From Psychometric Networks to Gaussian Graphical Models

The statistical model underlying many psychometric networks for continuous or ordinal data is the Gaussian Graphical Model (GGM). A GGM represents a set of random variables as nodes in a graph, where an edge between two nodes signifies that the variables are conditionally dependent, given all other variables in the network. Conversely, the absence of an edge implies conditional independence. These conditional (in)dependencies are encoded in the precision matrix (Ω), which is the inverse of the variance–covariance matrix (Σ). Specifically, a zero entry in the off-diagonal of the precision matrix, Ω*_ij_* = 0, corresponds to the absence of an edge between nodes *i* and *j*. The edges in a GGM are typically weighted by partial correlation coefficients, which quantify the association between two variables after controlling for the influence of all other variables in the model (Epskamp et al., 2017b). This ability to distinguish between direct and indirect associations is a key advantage of GGMs over simple correlation networks. While a correlation matrix shows marginal associations, which can be dense and difficult to interpret, a GGM aims to reveal a sparser, more direct structure of relationships.

### 1.2. The Cross-Disciplinary Utility of Gaussian Graphical Models

The challenge of inferring conditional dependence structures from multivariate data is common to several scientific disciplines. In biostatistics and genomics, for instance, GGMs are used to infer gene co-expression networks from high-dimensional RNA-Seq data. In this context, nodes represent genes, and an edge suggests a direct regulatory relationship, helping to identify biological pathways and disease susceptibility genes (Zhao & Duan, 2019). Similarly, GGMs have been applied to reconstruct protein–protein interaction networks and metabolite association networks, providing a system-level view of biological processes (Rual et al., 2005). In health sciences, network analysis is used to model complex interactions between symptoms, social factors, and health behaviors, offering insights for targeted interventions (Leme et al., 2020). The statistical challenges—such as dealing with high dimensionality, selecting an appropriate model, and determining the required sample size—are shared across these fields, making interdisciplinary dialogue essential for methodological advancement.

### 1.3. The Landscape of Network Estimation Algorithms

In practice, the true precision matrix is unknown and must be estimated from data. Because the number of potential parameters (edges) grows quadratically with the number of nodes, a key challenge is to avoid overfitting and identify a sparse, interpretable network. Several algorithms have been developed for this purpose, the choice of which depends on the data characteristics and the research objectives. Some alternatives are:

- The Ising Model. For binary data (e.g., present/absent symptoms), the Ising model, originating from statistical physics, is the appropriate analogue to the GGM. It is equivalent to a log-linear model with only pairwise interactions and can be estimated using penalized logistic regression (Marsman et al., 2018).
- Mixed Graphical Models (MGMs). Real-world datasets in health and social sciences often contain a mix of variable types (e.g., continuous, categorical, count). MGMs extend the graphical modeling framework to handle such heterogeneous data, estimating a single network of conditional dependencies across different variable domains (Altenbuchinger et al., 2020).
- EBICglasso. The graphical LASSO (glasso, Friedman et al., 2008) is a penalized maximum likelihood method that uses an l1 penalty to shrink small partial correlations to exactly zero. The magnitude of this penalty is controlled by a tuning parameter, *λ*. To select the optimal model from the set of networks estimated with different *λ* values, the Extended Bayesian Information Criterion (EBIC, Foygel & Drton, 2010) is often employed. EBIC (Chen & Chen, 2008) modifies the standard BIC by adding a hyperparameter (ranging from 0 to 1), which tunes the penalty for model complexity. Higher values of *γ* impose a stronger penalty on denser networks, favoring sparser models and helping to control the false positive rate. This combined procedure is commonly known as EBICglasso (Epskamp, 2017), and has been a dominant approach in psychometrics (Isvoranu & Epskamp, 2023).
- Alternative GGM Estimators: The dominance of EBICglasso is not without critique. Some research suggests that in the low-dimensional settings (*p* ≪ *n*) common in psychology, its advantages diminish, and it may be outperformed by other methods. Non-regularized methods, based on multiple regression with stepwise selection or bootstrapping, have been proposed as alternatives that can offer better control over false positives (Williams et al., 2019). Furthermore, Bayesian methods for GGM estimation provide a framework for quantifying uncertainty about both the network structure and its parameters (Franco et al., 2024).

EBICglasso shows a proper performance for estimating models with ordinal and continuous models (Epskamp, 2017), being used as a tool for both exploratory (Epskamp et al., 2018b; Golino & Epskamp, 2017) and confirmatory (Epskamp et al., 2017b) analysis. Although these developments provide new possibilities for studying the inner structure of psychological assessment devices, their recent emergence deals with aspects that generate no consensus or are scarcely studied. One of these is the sample size required for making reliable estimations. There is consensus in posing that the greater the sample size, the more reliable and accurate the network; however, there are no precise recommendations about the proper size (de Ron et al., 2022; Epskamp, 2017; Epskamp et al., 2017a, 2018a; Johal & Rhemtulla, 2023). Some studies propose using simulated data to establish the sample size required for estimating a psychometric network (M. A. Constantin et al., 2023). This method requires defining the network structure or one of its features a priori and establishing a target value for a statistic index (e.g., 0.6 sensitivity) and an achievement criterion (e.g., 0.8 probability for reaching 0.6 sensitivity). The authors implement this method in powerly library for R studio (M. Constantin, 2022); however, it currently supports only four target statistics (sensitivity, specificity, Matthews correlation, and Pearson correlation). Although it uses EBICglasso for estimating networks during the simulation process, it does not allow manipulating parameter *γ*. So, in practice, it compels the researcher to estimate the model with *γ* = 0.5.

Literature does not particularly recommend the use of specific values of *γ*. However, it makes general recommendations for its use. For example, Chen and Chen (2008) recommend values of *γ* = 0.5 because it provides a good equilibrium between fit and simplicity. Foygel and Drton (2010), concerning high dimensionality models and a few observations, recommend the use of values close to 1 to favor simple networks and avoid weak or spurious connections. These authors pose that values between 0.25 and 0.50 provide a proper balance for most conditions.

### 1.4. The Present Study

As mentioned above, the EBICglasso algorithm demonstrates good performance and remains a de facto standard in applied psychometric research. However, evidence of its performance under different conditions of sample size, network complexity, and hyperparameter tuning is still scarce. Previous work, such as Epskamp (2017), has explored the impact of sample size and *γ*, but the influence of the number of nodes (*k*) and its interaction with other factors has been less studied.

This study seeks to address this shortcoming by evaluating the performance of the EBICglasso algorithm under a wide range of conditions, manipulating sample size (*n*), the number of nodes (*k*), and the EBIC hyperparameter *γ*. By evaluating the accuracy of network structure recovery, edge weight estimation, and centrality indices, we aim to provide clear, data-driven recommendations for applied research, helping them design more robust studies and make more reliable inferences from psychometric network models.

Given the scarce literature, specific hypotheses cannot be posed; however, some exploratory hypotheses will be formulated here. First, higher values of gamma hyperparameter (*γ*) are expected to increase the network estimation specificity and decrease its sensitivity, while intermediate values of gamma (e.g., 0.25–0.50) offer a suitable equilibrium between edge penalization and detection. Second, by increasing sample size (*n*), the estimation accuracy of the network structure is expected to improve, minimizing bias in estimating weight edge, and optimizing estimated centrality index accuracy. Third, network size (*k*, the number of nodes) will influence the estimation accuracy since a greater number of variables quickly increases the number of parameters to be estimated. This can pose an inherent challenge for network estimation accuracy. Finally, concerning interactions among the three variables in the design, the interaction between sample size (*n*) and network size (*k*) is expected to cause a predominant effect on estimation accuracy. This is because the combination of a limited sample size and a big number of nodes poses a methodological challenge well known in the estimation of high-dimensional models. In addition, the interaction between gamma hyperparameter (*γ*) and sample size (*n*) is expected to be relevant because of the impact both can have on estimation dispersion and the presence of spurious edges under different sample sizes.

## 2. Materials and Methods

To obtain a representative psychological network structure, the procedure followed by Epskamp (2017) was replicated. He used the Big Five Inventory (BFI) dataset available at psych library (Revelle, 2022) in R language version 4.4.2 (R Core Team, 2024). This dataset contains 2800 observations in 25 6-point Likert scale items assessing the five-factor personality model. Epskamp (2017) obtained the network structure by calculating the partial correlation coefficients of the sample. To create a disperse network, the absolute edge weights below 0.05 were set to 0. From this network, he simulated normal multivariate and ordinal data for six sample sizes: 50, 100, 250, 500, 1000 y 2500, making 1000 replicates for each size and type of variable. Epskamp (2017) generated ordinal data by means of random threshold sampling for each standard normal distribution variable and, then, he used these thresholds for discretizing each variable. In each replicate, he estimated the network using EBICglasso function from the qgraph package (Epskamp et al., 2012), hyperparameter gamma having five values: 0; 0.25; 0.50; 0.75; and 1.

Here, the study of Epskamp (2017) is extended to include network size as a condition. The dataset BFI is used for obtaining networks of 25, 20, 15, 10, and 5 nodes. The biggest network is the one used by Epskamp (2017). For each variable, the correlation average of the variable with the other variables belonging to the same factor (${\bar{r}}_{within}$); the correlation average of the variable with the variables belonging to the other factors (${\bar{r}}_{between}$); and the ratio between both averages (${\bar{r}}_{within}/{\bar{r}}_{between}$) were calculated. By eliminating the variable with the lowest value in this ratio in each dimension, a new dataset of 20 variables was created. This procedure was repeated to get datasets of 15, 10, and 5 variables, obtaining five conditions for the network size: 25, 20, 15, 10, and 5. The procedure of Epskamp (2017) was replicated in each of these datasets to obtain the network structure, simulate normal and ordinal data for different sample sizes, and estimate networks by varying hyperparameter *γ*. Unlike Epskamp (2017), the conditions of 50 observations were not considered here because they correspond to a little realistic condition in research practice since it makes design unnecessarily complex. A total of 1000 replications were made for each combination of network size, sample size, and type of variable. This, combined with the values of *γ*, generated 250,000 estimated networks. Following Skrondal (2000), the same random seed was used for starting each sequence of 1000 replicates to generate multivariate normal data, control randomness to ensure replicability, and reduce variance among runs due to randomness.

Following Hoekstra et al. (2024), indices were calculated for each estimated network to assess the estimated network accuracy to reproduce the true network structure. Sensitivity and specificity were calculated as measures of the estimated network accuracy. The estimation bias of both the entire network and only the true edges were calculated as accuracy measures of the estimated edges^1^. The correlation between the indices of the true and estimated network and the ratio of nodes correctly identified within 50% of the nodes with higher centrality indices were calculated as measures of centrality index accuracy. The centrality indices calculated were strength and expected influence. Table 1 shows the different conditions and dependent variables in this study.

## 3. Results

### 3.1. Invertible and Non-Empty Matrices

To estimate a network, the correlation matrices must be positive definite so that its inverse can be calculated. All matrices with continuous data met this condition, but 793 (3.17%) of the matrices with discrete data did not. Table 2 shows that the greater the ratio between sample size and the number of variables, the greater the number of positive definite matrices. Also, the percentage of invertible matrices is higher than 95% if this ratio is greater than 6.

Table 3 shows the percentage of non-empty networks (with one edge different than 0, at least) estimated for all the values of gamma and number of variables used. The percentage was calculated over the number of simulated samples, not the number of invertible matrices. Only sample sizes (n) with a combination of *γ* and *k* presenting a percent lower than 100% were included. Continuous variables show a similar pattern, although percentages are lower for *k* = 5 and higher for the other cases (see Supplementary Table S1).

The percentage of non-empty matrices increases under all network size conditions as the value of gamma decreases and sample size increases. In smaller networks, greater sample sizes are required to obtain non-empty networks. In bigger networks (*k* ≥ 10), on the other hand, non-empty networks are obtained with smaller sample sizes, exceeding 98% of non-empty matrices if the ratio between sample and matrix size (*n*/*k*) is 10 or higher for all values of *γ*. Therefore, although reducing the number of variables helps to obtain an invertible matrix, it negatively affects the network estimation and compels the use of higher sample sizes, some of which could be unviable in some contexts.

### 3.2. Effect of Simulated Conditions on Network Indicators

To analyze how different design features influence the quality of psychometric network estimations, three types of indicators were assessed: estimated network accuracy, edge estimation accuracy (i.e., links among variables) and centrality index accuracy, the latter making it possible to identify the most relevant nodes in the network structure. Mixed models were estimated to quantify the variance in these indicators explained by the type of data (*d*) (ordinal or continuous), the number of variables in the network (*k*), the value of hyperparameter gamma (*γ*), and sample size (*n*). Following Skrondal (2000), both main effects and two-factor interactions were considered since higher-order interactions tend to be difficult to interpret.

All the dependent variables presented positive asymmetry. So, they were transformed to approach them to a normal distribution. The standardized normal score (Cronbach, 1990) was used to transform sensitivity, specificity, and the proportion of nodes correctly identified within the top 50% of the nodes with higher strength and expected influence indices. According to Cronbach, the standardized normal score is calculated in two steps: First, the raw score is transformed into a percentile and, then, the latter is replaced by the corresponding standardized normal score. Regardless of the original variable distribution, the standardized normal score will approach the normal distribution owing to the relationship between the percentile and z. Since sensitivity, specificity, and proportion are proportions in themselves, they are transformed directly into z. This transformation renders results equal to or higher than others commonly used in proportions or probabilities such as logit or arccosine. The natural logarithm was used for transforming the estimation bias of the entire network and the one only for true edges. Transformation z from Fisher (1936) was used to transform the correlations between the strength and expected influence indices of the true and estimated network.

All the main effects and two-factor interactions were statistically significant (*p* < 0.001). Table 4 shows the explained variance ratio (partial ω^2^) for each independent variable, with values exceeding 0.14 highlighted to indicate a large effect (Lakens, 2013). The results show that sample size (*n*) is the most dominant factor, explaining the largest proportion of variance for nearly all performance indicators. The interaction between number of nodes and sample size (*k×n*) also consistently emerged as critical determinants of estimation quality. In contrast, the type of data (continuous vs. ordinal) had a minimal impact.

### 3.3. Estimated Network Accuracy

**Figure 1:**
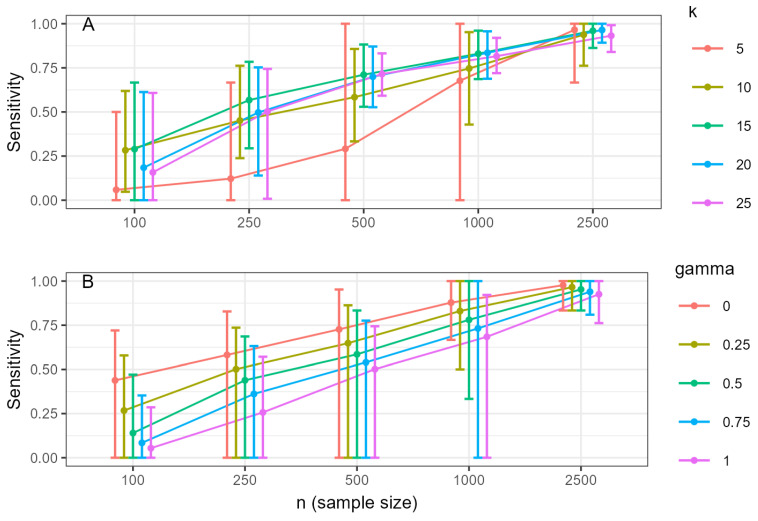
Sensitivity, according to (**A**) number of variables (*k*) and sample size and (**B**) value of gamma and sample size. Note: Error bars correspond to percentiles 2.5 and 97.5, while the circle corresponds to the mean.

The estimated network accuracy was assessed with two indicators: sensitivity and specificity. Sensitivity is defined as the ratio of true connections (edges) correctly identified in the estimated network. Figure 1 shows the effects of the two-factor interaction *k × n* (A) and *n × γ* (B) on sensitivity. Results show that sample size has a large effect on sensitivity. For any given network size or *γ* value, increasing the sample size led to substantial gains in sensitivity. This effect was more pronounced for larger, more complex networks, which require more data to be accurately recovered. The hyperparameter *γ* had a noticeable effect only at smaller sample sizes (*n* ≤ 250), where higher values of *γ* decreased sensitivity. With large samples (*n* ≥ 1000), the influence of *γ* became negligible, and sensitivity was consistently high. An exception was observed for very small networks (*k* = 5), which exhibited poor sensitivity even with large samples, suggesting that their sparse structure is difficult to recover.

**Figure 2:**
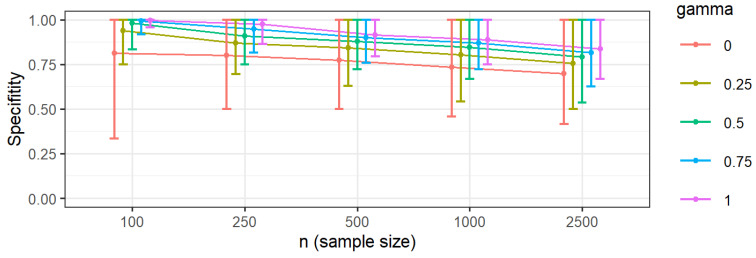
Specificity, according to value of gamma and sample size. Note: Error bars correspond to percentiles 2.5 and 97.5, while the circle corresponds to the mean.

Specificity, the model ability to avoid false connections in the estimated network, showed an inverse relationship with sample size and a positive relationship with *γ* (Figure 2). Larger samples and lower *γ* values lead to denser estimated networks, increasing the risk of including spurious connections (false positives). Even at the highest penalty (*γ* = 1), specificity could fall below 75% in large samples (*n* ≥ 1000), highlighting a trade-off between detecting true edges and avoiding false ones.

These findings reinforce the importance of sample size and the number of variables as determining factors for the ability of the model to recover the true network structure. Although parameter gamma plays a relevant modulating role, its influence weakens considerably as sample size increases. These results have direct implications in designing applied studies because they allow anticipating the conditions necessary for obtaining reliable estimations more accurately. Indeed, having enough observations and a suitable network size allows obtaining more accurate estimations, avoiding spurious connections, and improving both the model sensitivity and specificity.

### 3.4. Edge Weight Estimation Accuracy

**Figure 3:**
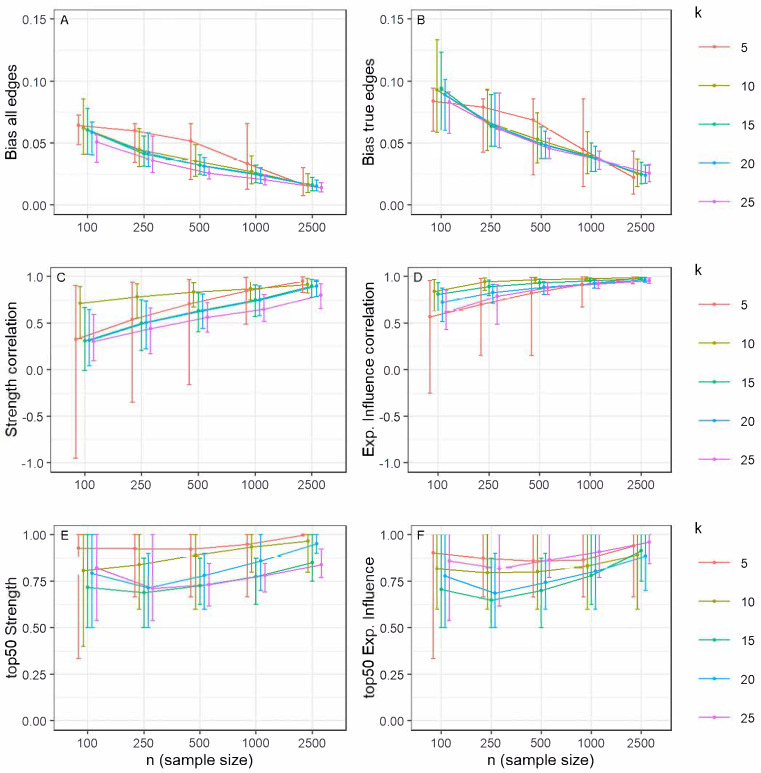
Network bias, centrality index correlations, and ratio of nodes correctly identified within the top 50% of centrality indices, according to number of variables (*k*) and sample size. (**A**) Bias for all edges, (**B**) bias for true edges, (**C**) node strength correlation, (**D**) node expected influence correlation, (**E**) ratio of nodes correctly identified within the top 50% of strength, and (**F**) ratio of nodes correctly identified within the top 50% of expected influence. Note: Error bars correspond to percentiles 2.5 and 97.5, while the circle corresponds to the mean.

The accuracy of the estimated edge weights was assessed via bias, calculated for all possible edges and for true edges only. For both measures, bias systematically decreased as sample size increased, confirming that more data leads to more precise estimates (Figure 3A,B). This effect was most pronounced in larger networks (*k* ≥ 10), which not only showed lower average bias but also less dispersion in the estimates, indicating greater stability. The bias calculated only on true edges was generally higher and more variable than the overall bias, especially at small sample sizes. However, with sufficient data (*n* ≥ 500), bias became consistently low across nearly all conditions. The effect of *γ* on bias was minimal, suggesting that under adequate sample conditions (*n* ≥ 500 and *k* ≥ 10), EBICglasso recovers the magnitude of relationships between the network nodes quite accurately.

### 3.5. Centrality Index Accuracy

The centrality index estimation accuracy was assessed from two complementary measures: the correlation between the centrality estimated and true values and the ratio of nodes correctly identified within the top 50% in each index. Two centrality indices widely used in psychometric networks were used: strength and expected influence.

The correlation for strength centrality improved systematically with sample size (Figure 3C). In small networks (*k* = 5) with few observations (*n* ≤ 250), the estimates were highly unstable, sometimes even yielding negative correlations. However, in larger networks (*k* ≥ 10), the correlations were more stable and consistently high, especially when the sample-to-variable ratio (*n*/*k*) was 10 or greater.

Expected influence proved to be a more robust index (Figure 3D). Its correlations with the true values were generally higher and less dispersed than those for strength across all conditions. Even in smaller networks, reliable estimates were achieved with moderate sample sizes (*n* ≥ 500). For networks with 10 or more nodes, high correlations were obtained even with smaller samples, provided the *n*/*k* ratio was at least 10.

In general, the ratio of correctly identified central nodes shows values higher than 60%, steadily increased with both sample size and network size for both indices (Figure 3E,F), but expected influence behaves as a more stable index than strength. Together, these findings indicate that centrality indices can be estimated reliably when the network is sufficiently large (*k* ≥ 10) and the sample size is adequate, with an *n*/*k* ratio of at least 10 serving as a good heuristic for achieving stable results.

## 4. Discussion

In this study, the joint effect of sample size, the type and number of variables, and hyperparameter gamma on psychometric network estimation if using EBICglasso algorithm is assessed. The main objective was identifying the minimal sample size required for making reliable estimations, considering different values of gamma and the number of variables (or items). Our results highlight the importance of sample size, its interaction with network size, and the modulating role of the *γ* hyperparameter.

### 4.1. Main Findings

Results show that the main determinant of estimation accuracy across nearly all metrics (including sensitivity, edge weight bias, and centrality stability) is sample size (*n*), and that it is moderated by the number of nodes (*k*) in the network. Our findings suggest that the sample-to-variable ratio (*n*/*k*) is a more practical heuristic than absolute sample size. A ratio of at least 10 cases per variable (*n*/*k* ≥ 10) proved to be a robust threshold for both achieving invertible correlation matrices with ordinal data and for obtaining reliable estimates of centrality indices.

The EBICglasso algorithm seeks to control spurious correlations by equating the weakest to zero, thus obtaining a more disperse network than the initial partial correlation matrix. For this purpose, it uses hyperparameter gamma, penalizing the model complexity and partly controlling the estimated network dispersion. Therefore, this dispersion greatly depends on sample size and network size and, to a lesser extent, on gamma. Most small networks (five nodes), are empty or extremely dispersed, making estimation more erratic. Thus, avoiding them is recommendable. Hereinafter, networks between 10 and 25 nodes will be referred to.

In controlling the estimated network density, gamma indirectly affects the network estimation accuracy. Although results here indicate that the impact of gamma is generally smaller than that of sample and network size, it cannot be ignored. In describing the behavior of two indices assessing the accuracy of the estimated network to reproduce the global structure of the true network, as gamma increases, so does specificity (ratio of edges absent in the true network, which are also absent in the estimated network). In other words, as the estimated network presents fewer edges, these tend to coincide with the true ones. On the other hand, as gamma increases, sensitivity (ratio of edges present in the true network, which are also present in the estimated network) decreases. That is, the fewer the edges present in the estimated network, the fewer the true edges detected, although this is expected and does not mean that the estimated network is considerably far from the true one. When sample size is medium or high (*n* > 250), the greater network density mitigates this problem. If a researcher is interested in the strongest network edges and/or wants to avoid false positives, medium-high values of gamma (*γ* ≥ 0.5) must be set. If *n* = 2500, the estimated network will probably be denser than the true one. So, false positives will not be completely avoided (Epskamp et al., 2017a). On the other hand, for 500 ≤ *n* ≤ 1000, the estimated network density will be closer to the true one, thus decreasing false positives. Finally, if *n* ≤ 250, false positives will be minimal, but at the expense of obtaining networks considerably more disperse than the true one.

The behavior of two bias indices shows that the estimation bias depends more on both the sample and network size than on the value of gamma. As a whole, bias is not a concern in networks with *n* ≥ 100 and *k* ≥ 15. As the value of gamma increases, the calculated bias slightly increases only for true edges, while it slightly decreases for all edges. If *n*/*k* > 10, both biases will be low. In addition, the differences of these indicators among the various values of gamma and sample sizes are small. So, estimated values can be considered as close to true values, as a whole.

To assess centrality index accuracy, the correlation between the calculated indices of the estimated networks and those calculated for true networks, along with the proportion of nodes correctly detected within the top 50% were used. Although strength shows negative correlations, these are scarce if *n*/*k* ≥ 10 and non-existent if *n* ≥ 500. As to expected influence, correlations are always non-negative and moderate or high if *n*/*k* ≥ 10. Both in strength and expected influence, the ratio of edges correctly detected within the top 50% is usually higher than 0.5 if *n*/*k* ≥ 10. Additionally, as sample size and the number of variables increase, the mean of this ratio increases, while its variability decreases. A similar behavior was observed in bridge centrality indices (Supplementary Figure S2). However, the greater dispersion shown makes sample sizes greater than 250 and with a *n*/*k* ≥ 20 ratio recommendable.

### 4.2. Comparison with Existing Literature

Our findings corroborate and extend previous research in network psychometrics. Epskamp’s (2017) simulation study, on which our work is based, also identified sample size as a key factor for accurate estimation and described the behavior of *γ* in controlling network density. Our results are consistent with his general conclusions. However, by introducing the number of nodes (*k*) as a manipulated variable, our study reveals significant interaction effects. We show that the sample size required for a given level of accuracy is not fixed but depends on the complexity of the model being estimated. The emergence of the n/k ratio as a practical guide is a key contribution that goes beyond a simple recommendation for “large samples.”

Furthermore, our results are consistent with the findings of Isvoranu and Epskamp (2023), who identify a trade-off between model discovery (sensitivity) and false positive control (specificity) when comparing several estimation algorithms. Our finding that EBICglasso can produce denser-than-real networks with very large samples, thereby reducing specificity, aligns with concerns raised by Epskamp et al. (2017a). This reinforces the idea that no estimation method or parameter setting is optimal for all research objectives; the choice should be based on whether the researcher wishes to prioritize discovery or confirmatory rigor.

### 4.3. Limitations and Future Directions

This study is not without limitations. First, although the true network was defined from the real data of an attribute well known in psychology, only one original matrix, that was iteratively reduced to obtain the smaller ones, was used. This method for generating networks of varying sizes may lead to structures that are not fully representative of the diversity of constructs encountered in applied research and could result in smaller networks that are artificially sparse or have altered community structures. Instead, different instruments and/or constructs of different sizes could be used, thus increasing the ecological validity of results. Future simulation studies should employ more sophisticated and ecologically valid data-generating mechanisms. One approach is to simulate data from known factor model structures, which would allow for a direct comparison of how network and latent variable models recover a known true model (Christensen & Golino, 2021). Another approach is to generate networks based on established topological models, such as scale-free or small-world networks (Hoekstra et al., 2024).

Second, all ordinal variables were simulated at five levels. While common in psychology, this does not cover the full range of data types that researchers may address. Future work should extend this research to include binary data, which would require a comparative evaluation of Ising model estimators, and mixed data types (e.g., combinations of continuous, categorical, and count variables), which would require the use of graphical mixed models (GMMs).

Third, this study focused exclusively on the EBICglasso algorithm. Although it is a widely used method, it is crucial for the field to continue the comparative evaluation of different estimators. Methods such as non-regularized GGM estimation (Williams et al., 2019) or Bayesian approaches (Huth et al., 2025) may offer advantages in certain contexts, such as the low-dimensional settings often found in psychology, by providing better control over false positives or more intuitive quantification of uncertainty. Comprehensive simulation studies comparing these alternatives is needed for developing reliable methodological guidelines.

Finally, from the different parameters that can be modified in the EBICglasso function of qgraph package, only gamma was. Hence, the eventual effects of other EBICglasso parameters on the indicators assessed here are unknown.

### 4.4. Practical Recommendations and Conclusions

In the light of these findings, some recommendations can be made for applied researchers planning to use EBICglasso for network estimation:

Prioritize sample size based on network complexity. Sample size is the most determining factor to obtain accurate estimations of the network and its centrality indices. A ratio of at least 10 cases per variable (*n*/*k* ≥ 10) is recommended to ensure invertible matrices and obtain reliable results for most conditions. Also, to interpret the most relevant nodes reliably, sufficient sample sizes and properly sized networks are required. Results here suggest that an *n*/*k* ≥ 10 ratio is a good starting point to obtain satisfactory centrality estimations (*n*/*k* ≥ 20 bridge centrality), expected influence being a more robust index than strength.

Avoid very small networks. Networks with fewer than 10 nodes tend to produce unstable and erratic estimates and show low sensitivity. Whenever possible, researchers should aim to model networks with 10 or more variables to achieve a level of structural complexity sufficient for stable estimation.

Tune the *γ* hyperparameter to your research goal: The choice of *γ* should be a deliberate decision based on the study’s aims.

- For exploratory research where the goal is to discover potential connections and maximize sensitivity, use lower values of (*γ* ≤ 0.25).
- For confirmatory research or studies where the priority is to minimize false positives and identify only the most robust connections, use higher values (*γ* ≥ 0.5).
- Be aware that the impact of *γ* decreases substantially in very large samples (*n* ≥ 1000).

To our knowledge, this study is the first explicitly exploring the minimal sample size required for obtaining reliable estimations in network psychometry, considering different values of gamma and the number of variables. The effect of the combination of gamma, sample size, and number of variables on various indices assessing estimation accuracy is explored in three aspects: the reproduction of the global structure of the true network, the true weights of edges, and centrality indices. In addition, continuous and ordinal variables were simulated, this being the measurement level typical of psychological instruments. Hence, the results here described can be useful guidelines for an applied researcher to plan data collection and/or evaluate the reliability of his/her results.

## Figures and Tables

**Table 1:** Independent and dependent variables.

**Independent Variables**	**Values**
Type of data (*d*)	Continuous
	Ordinal
Sample size (*n*)	100
	250
	500
	1000
	2500
Network size (number of nodes) (*k*)	5
	10
	15
	20
	25
Value of gamma (*γ*)	0.00
	0.25
	0.50
	0.75
	1.00
**Dependent Variables**	**Evaluation Metric**
Estimated network accuracy	Sensitivity
	Specificity
Edge weight estimation accuracy	Estimation bias for all edges
	Estimation bias for true edges
Centrality index accuracy	Correlation between the true and estimated node strength
	Ratio of nodes correctly identified within the top 50% in strength
	Correlation between the true and estimated node expected influence
	Ratio of nodes correctly identified within the top 50% in expected influence

**Table 2:** Percentage of positive-definite matrices, according to the number of variables (*k*) and the sample size (*n*) of ordinal data.

*k*\*n*	100	250	500	1000	2500
5	99.9	100	100	100	100
10	99.7	99.9	100	100	100
15	96.5	99.9	100	100	100
20	82.7	99.9	100	100	100
25	42.8	99.4	100	100	100

**Table 3:** Percentage of non-empty networks, according to number of variables (*k*), sample size (*n*) and gamma (*γ*) in discrete variables.

*k*	*n*\*γ*	0	0.25	0.50	0.75	1
5	100	52.2	35.1	25.8	19.8	15.8
	250	67.3	52.2	40.8	32.1	26.2
	500	86.4	75.4	62.2	52.2	43.6
	1000	99.6	98.8	94.4	88.1	79.5
	2500	100.0	100.0	100.0	100.0	99.8
10	100	99.7	99.7	99.7	99.1	95.5
	250	99.9	99.9	99.9	99.9	99.9
15	100	96.5	96.5	95.5	85.5	64.8
	250	99.9	99.9	99.9	99.9	99.9
20	100	82.7	82.3	70.4	45.0	27.8
	250	99.9	99.9	99.9	99.9	99.9
25	100	42.8	41.3	23.6	13.4	8.0
	250	99.4	99.4	99.4	99.4	98.2

**Table 4:** Partial ω² of the main effects and interactions for type of variable (*d*), number of variables (*k*), value of gamma (*γ*), and sample size (*n*).

	(1) Sensitivity	(2) Specificity	(3) Bias of All Edges	(4) Bias of True Edges	(5) r Strength	(6) r Expected Influence	(7) Top-50% Strength	(8) Top-50% Expected Influence
*d*	*0.001*	*0.066*	*0.103*	*0.059*	*0.072*	*0.097*	*0.004*	*0.005*
*k*	**0.412**	**0.217**	**0.483**	0.060	**0.442**	**0.539**	**0.166**	**0.439**
*γ*	**0.434**	**0.299**	**0.331**	**0.352**	0.119	**0.141**	0.010	0.025
*n*	**0.915**	**0.392**	**0.927**	**0.914**	**0.701**	**0.779**	**0.216**	**0.341**
*d × k*	0.002	*0.003*	0.009	0.008	0.015	*0.003*	0.004	0.006
*d × γ*	*0.002*	0.011	0.026	0.008	*0.009*	0.011	0.003	*0.001*
*d × n*	0.025	0.007	0.041	0.030	0.015	0.009	*0.001*	0.001
*k × γ*	0.050	0.020	*0.004*	*0.003*	0.087	0.012	0.024	0.047
*k × n*	**0.352**	0.089	**0.215**	**0.209**	**0.173**	**0.154**	**0.146**	**0.203**
*n × γ*	**0.219**	0.134	0.020	0.015	0.015	0.015	0.004	0.010

Note: Numbers in **bold** indicate $\omega_{p}^{2}>0.14$ for main effects and interactions. Numbers in *italics* indicate the lowest value.

## Data Availability

R scripts needed to replicate the whole process described in the Materials and Methods section and to obtain reported results in the Results section are included in Supplementary Material. Data required to obtain the results reported in the Results section are included in Supplementary Material in RData format.

## Note

1 The median absolute deviation (MAD) between the edge weights of the true network and the weight of the estimated network was used as a bias indicator.

## Supplementary Materials

The following supporting information can be downloaded at: https://www.mdpi.com/article/10.3390/ejihpe15110235/s1, Figure S1: Sensitivity according to number of variables (*k*) value of gamma and sample size; Figure S2: Ratio of nodes correctly identified within the top 20% of bridge centrality indices, bridge centrality index correlations, and ratio of nodes correctly identified within the top 50% of bridge centrality indices, according to number of variables (*k*) and sample size; Table S1: Percent of non-empty networks, according to number of variables (*k*), sample size (*n*) and gamma (*γ*) in discrete and continuous variables; Table S2: Partial ω² of the main effects and interactions for type of variable (*d*), number of variables (*k*), value of gamma (*γ*), and sample size (*n*) in bridge centrality indices; Table S3: Partial *η*² of the main effects and interactions for type of variable (*d*), number of variables (*k*), value of gamma (*γ*), and sample size (*n*).

## Abbreviations

The following abbreviations are used in this manuscript:

BFI	Big Five Inventory
