# Supplementary material for: Psychometric Network Model Recovery: The Effect of Sample Size, Number of Items, and Number of Nodes
Source: Eur J Investig Health Psychol Educ. 2025 Nov 18;15(11):235. doi: 10.3390/ejihpe15110235 (PMC12651093; doi:10.3390/ejihpe15110235)
Supplement: Supplementary file 1 [file ejihpe-15-00235-s001.zip › SupplementaryTable S1.pdf]

**Supplementary Table S1.** Percent of non-empty networks, according to number of variables (*k*), sample size (*n*) and gamma (*γ*) in discrete and continuous variables.

| | | **Discrete variables** | | | | | **Continuous variables** | | | | |
|---|---|---|---|---|---|---|---|---|---|---|---|
| *k* | *n \ γ* | *0* | *.25* | *.50* | *.75* | *1* | *0* | *.25* | *.50* | *.75* | *1* |
| *5* | 100 | 52.2 | 35.1 | 25.8 | 19.8 | 15.8 | 36.0 | 22.3 | 17.1 | 15.0 | 13.0 |
| | 250 | 67.3 | 52.2 | 40.8 | 32.1 | 26.2 | 54.8 | 37.3 | 27.1 | 21.9 | 18.9 |
| | 500 | 86.4 | 75.4 | 62.2 | 52.2 | 43.6 | 84.4 | 68.5 | 52.3 | 39.7 | 30.6 |
| | 1000 | 99.6 | 98.8 | 94.4 | 88.1 | 79.5 | 99.7 | 98.5 | 93.5 | 86.7 | 74.9 |
| | 2500 | 100.0 | 100.0 | 100.0 | 100.0 | 99.8 | | | | | |
| *10* | 100 | 99.7 | 99.7 | 99.7 | 99.1 | 95.5 | 100.0 | 100.0 | 100.0 | 100.0 | 99.0 |
| | 250 | 99.9 | 99.9 | 99.9 | 99.9 | 99.9 | | | | | |
| *15* | 100 | 96.5 | 96.5 | 95.5 | 85.5 | 64.8 | 100.0 | 100.0 | 99.1 | 86.2 | 60.0 |
| | 250 | 99.9 | 99.9 | 99.9 | 99.9 | 99.9 | | | | | |
| *20* | 100 | 82.7 | 82.3 | 70.4 | 45.0 | 27.8 | 100.0 | 99.5 | 88.1 | 61.3 | 36.5 |
| | 250 | 99.9 | 99.9 | 99.9 | 99.9 | 99.9 | | | | | |
| *25* | 100 | 42.8 | 41.3 | 23.6 | 13.4 | 8.0 | 100.0 | 95.6 | 70 | 43 | 24.6 |
| | 250 | 99.4 | 99.4 | 99.4 | 99.4 | 98.2 | 100.0 | 100 | 100 | 100 | 99.4 |

Note: Only rows with combinations of *γ* and *k* presenting a percent lower than 100% were included
