# Supplementary material for: Psychometric Network Model Recovery: The Effect of Sample Size, Number of Items, and Number of Nodes
Source: Eur J Investig Health Psychol Educ. 2025 Nov 18;15(11):235. doi: 10.3390/ejihpe15110235 (PMC12651093; doi:10.3390/ejihpe15110235)
Supplement: Supplementary file 1 [file ejihpe-15-00235-s001.zip › SupplementaryTable S2.pdf]

Supplementary Table S2. Partial ω² of the main effects and interactions for type of variable (*d*), number of variables (k), value of gamma (*γ*), and sample size (*n*) in bridge centrality indices.

	**(1) r bridge strength**	**(2) r bridge expected influence**	**(3) Top-50% bridge strength**	**(4) Top-50% bridge expected influence**	**(5) Top-20% bridge strength**	**(6) Top-20% bridge expected influence**
d	*.065*	*.057*	*.012*	*.002*	*.001*	*.006*
k	**.311**	**.217**	**.293**	**.162**	**.110**	**.257**
γ	**.225**	**.255**	.023	.069	.011	.006
n	**.805**	**.823**	**.398**	**.558**	**.218**	**.391**
d * *k*	*.003*	*.003*	*.004*	.001	.002	.005
d * *γ*	.009	.009	.000	*.001*	*.000*	*.001*
d * *n*	.025	.040	.004	.008	.007	.003
k * *γ*	.033	.012	.025	.010	.021	.005
k * *n*	**.108**	.063	.095	.089	.072	.085
n * *γ*	.016	.013	.002	.009	.008	.007

Note: Numbers in **bold** indicate $\omega_p^2 > 0.1$ for main effects and interactions. Numbers in *italics* indicate the lowest value.
