# Supplementary material for: Psychometric Network Model Recovery: The Effect of Sample Size, Number of Items, and Number of Nodes
Source: Eur J Investig Health Psychol Educ. 2025 Nov 18;15(11):235. doi: 10.3390/ejihpe15110235 (PMC12651093; doi:10.3390/ejihpe15110235)
Supplement: Supplementary file 1 [file ejihpe-15-00235-s001.zip › SupplementaryTable S3.pdf]

Supplementary Table S3. Partial η^2 of the main effects and interactions for type of variable (*d*), number of variables (k), value of gamma (γ), and sample size (*n*).

	(1) Sensitivity	(2) Specificity	(3) Bias of all edges	(4) Bias of true edges	(5) r strength	(6) r expected influence	(7) Top-50% strength	(8) Top-50% expected influence	(9) r bridge strength	(10) r bridge expected influence	(11) Top-50% bridge strength	(12) Top-50% bridge expected influence	(13) Top-20% bridge strength	(14) Top-20% bridge expected influence
d	*.001*	*.066*	*.103*	*.059*	*.072*	*.097*	*.004*	*.005*	*.065*	*.057*	*.012*	*.002*	*.001*	*.006*
k	**.412**	**.217**	**.483**	.061	**.442**	**.539**	**.166**	**.439**	**.311**	**.217**	**.293**	**.162**	**.110**	**.257**
γ	**.434**	**.300**	**.331**	**.352**	**.119**	**.141**	.010	.025	**.225**	**.255**	.023	.069	.011	.006
n	**.915**	**.392**	**.927**	**.914**	**.701**	**.779**	**.216**	**.341**	**.805**	**.823**	**.398**	**.558**	**.218**	**.391**
d * *k*	.002	*.003*	.009	.008	.015	*.003*	.004	.006	*.003*	*.003*	.004	.002	.002	.005
d * γ	*.002*	.011	.026	.008	*.009*	.011	.003	*.001*	.009	.009	*.000*	*.001*	*.000*	*.001*
d * *n*	.025	.007	.041	.030	.015	.009	*.001*	.001	.025	.040	.004	.008	.007	.003
k * γ	.050	.020	*.004*	*.003*	.087	.012	.025	.047	.033	.012	.025	.010	.021	.005
k * *n*	**.353**	.090	**.216**	**.210**	**.174**	**.154**	**.147**	**.203**	**.108**	**.063**	.095	.089	.073	.086
n * γ	**.219**	**.134**	.021	.015	.015	.015	.004	.011	.016	.014	.002	.009	.008	.007

Note: Numbers in **bold** indicate $\eta_p^2 > 0.1$ for main effects and interactions. Numbers in *italics* indicate the lowest value.
