# Supplementary figures and images for: Psychometric Network Model Recovery: The Effect of Sample Size, Number of Items, and Number of Nodes
Source: Eur J Investig Health Psychol Educ. 2025 Nov 18;15(11):235. doi: 10.3390/ejihpe15110235 (PMC12651093; doi:10.3390/ejihpe15110235)

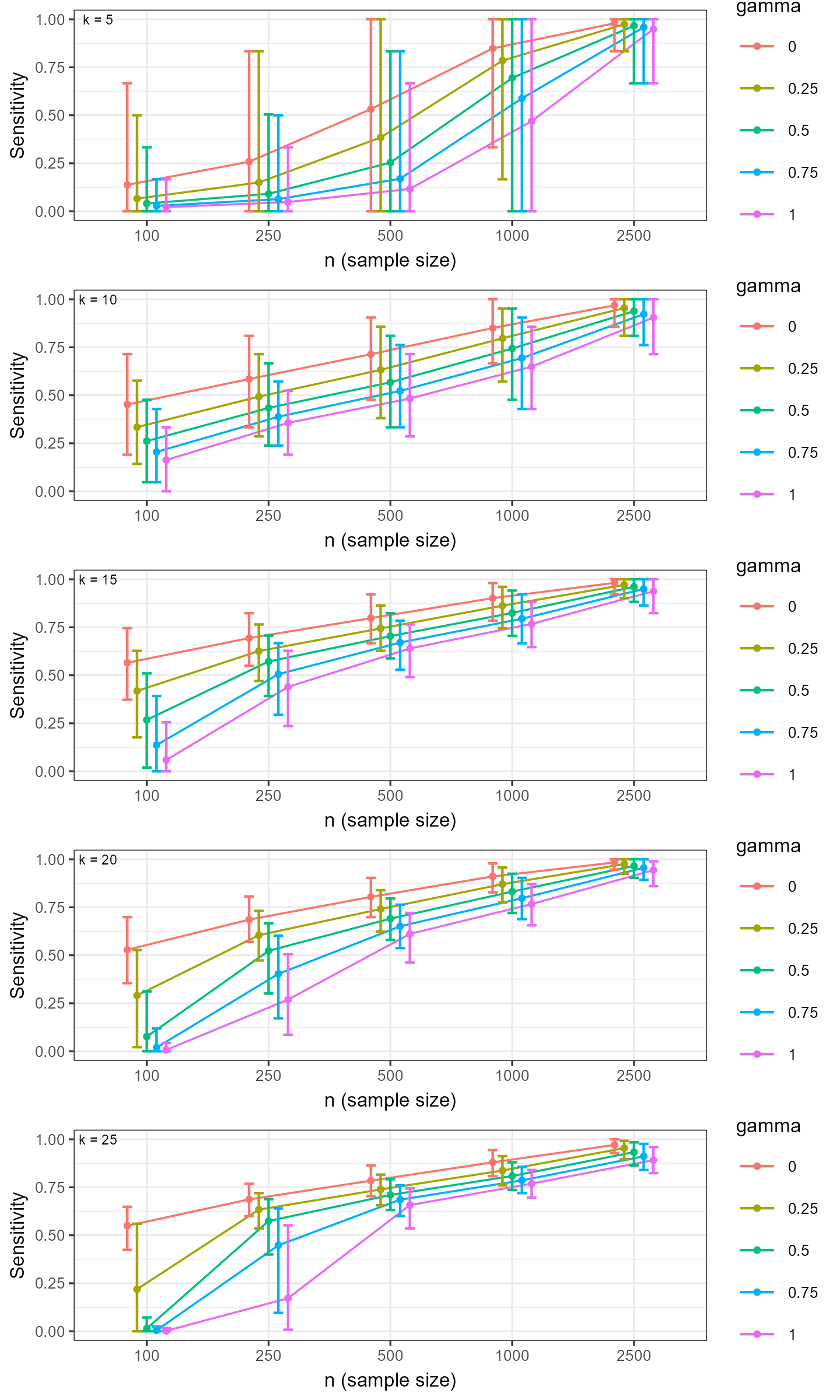

Supplement: Supplementary file 1 [file ejihpe-15-00235-s001.zip › figS01.png]

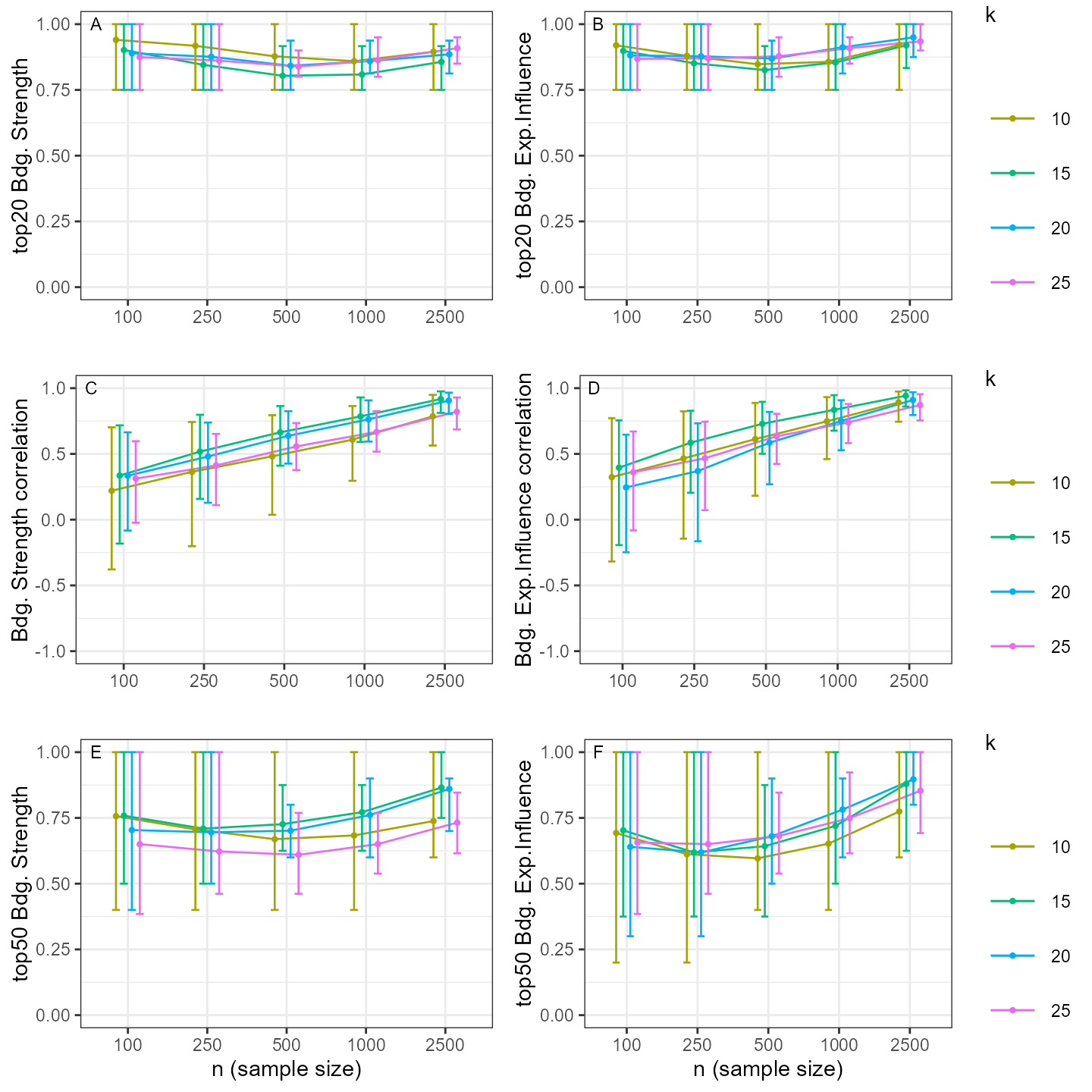

Supplement: Supplementary file 1 [file ejihpe-15-00235-s001.zip › figS02.png]
